# A Moveable Feast: Insects Moving at the Forest-Crop Interface Are Affected by Crop Phenology and the Amount of Forest in the Landscape

**DOI:** 10.1371/journal.pone.0158836

**Published:** 2016-07-06

**Authors:** Ezequiel González, Adriana Salvo, María Teresa Defagó, Graciela Valladares

**Affiliations:** Centro de Investigaciones Entomológicas de Córdoba–Instituto Multidisplinario de Biología Vegetal (CONICET–UNC), Córdoba, Argentina; The University of Sydney, AUSTRALIA

## Abstract

Edges have become prevailing habitats, mainly as a result of habitat fragmentation and agricultural expansion. The interchange of functionally relevant organisms like insects occurs through these edges and can influence ecosystem functioning in both crop and non-crop habitats. However, very few studies have focused on the directionality of insect movement through edges, and the role of crop and non-crop amount has been ignored. Using bi-directional flight interception traps we investigated interchange of herbivore, natural enemy, pollinator and detritivore insects between native forest fragments and soybean crops, simultaneously considering movement direction, forest cover in the landscape and crop phenology. In total, 52,173 specimens and 877 morphospecies were collected. We found that, within most functional and taxonomic groups, movement intensity was similar (richness and/or abundance) between directions, whereas a predominantly forest-to-crop movement characterized natural enemies. Insect movement was extensively affected by crop phenology, decreasing during crop senescence, and was enhanced by forest cover particularly at senescence. Mainly the same herbivore species moved to and from the forest, but different natural enemy species predominated in each direction. Finally, our analyses revealed greater forest contribution to natural enemy than to herbivore communities in the crop, fading with distance to the forest in both groups. By showing that larger amounts of forest lead to richer insect interchange, in both directions and in four functional groups, our study suggests that allocation to natural and cultivated habitats at landscape level could influence functioning of both systems. Moreover, natural enemies seemed to benefit more than pests from natural vegetation, with natural enemy spillover from forests likely contributing to pest control in soybean fields. Thus consequences of insect interchange seem to be mostly positive for the agroecosystem, although consequences for the natural system deserve further study.

## Introduction

Agricultural expansion drives loss and fragmentation of natural ecosystems, creating large expanses of abrupt boundaries between crop and non-crop habitats. As a result, edges are becoming increasingly common habitats [1]. The interchange of organisms between adjacent habitats occurs through these edges and is influenced by their contrast (i.e. how different the adjacent habitats are) [2], shape [3,4], crop phenology [1] and other characteristics of the landscapes and species involved (such as habitat patchiness or matrix quality) [5].

Insects are an important taxonomic group for agriculture, including herbivore species and among them some of the most harmful crop pests, while on the other hand, contributing a wide variety of natural enemies that help controlling pest populations [6]. Pest control provided by natural enemy insects, including parasitoids and predators, is an essential ecosystem service valued in $400 billion per year worldwide [7]. In addition to herbivores and natural enemies, insects provide indispensable pollinators for more than two-thirds of the world´s cultivated species [8]. The value of the pollination ecosystem service has been estimated to be between $5000 and 14000 million per year in the US alone [9,10], from which a large proportion is attributed to native insect pollinators [11]. Additionally, detritivore insect species are relevant for crops because of their contribution to nutrient cycling [6]; services provided by dung beetles alone may amount to $380 million per year [11].

Many species within these functional groups depend on the presence of natural ecosystems to complete their cycles [12,13]. Consequently, the proximity and amount of natural vegetation in the landscape can influence insect richness and abundance in agroecosystems [14–16]. It is well known that insect biodiversity in crops is enhanced by the presence of non-crop habitats in the landscape [15], but whether this is the result of improved interchange between habitats remains unknown.

Insect movement between crop and non-crop habitats can affect ecosystem functioning at both sides of the boundary (functional spillover) [17]. For the productive land, these changes can have either positive (e.g. natural enemies contributing to reduce pest populations) [18] or negative (when herbivores arrive to crops and produce economic damage) consequences [19]. Similar outcomes might occur in natural habitats, however this topic has been much less studied [17]. Therefore, it is important to know which functional groups of insects are involved in crop / non-crop interchange and how this process is influenced by landscape structure or temporal changes.

The predominant direction of movement between natural and cultivated habitats is expected to follow resource availability and thus to change with the phenological status of the crop, with more herbivores and natural enemies moving to the crop after sowing and from the crop to the natural habitat after harvest [1]. The scarce evidence available supports the expectation of early non-crop to crop movement for herbivores [20,21]. However, emigration of natural enemies from the crop seems to be continuous throughout its cycle, rather than a post-harvest peak [22]. For pollinators, movement in both directions is expected to be most intense during crop flowering. Moreover, since most species need natural habitats to complete their cycles, pollinators should move between both habitats within short periods of time [17]. As for detritivores, their interchange between natural and cultivated habitats has received little attention, but being mostly ground-dwelling organisms and considering the disturbances typical of agricultural soils, natural vegetation patches could act as refuges for these insects. Movement of dung and carrion beetles between natural and managed habitats seems to depend on edge permeability, with an intense interchange when both habitats have a similar structure [23] and a restricted movement for contrasting, hard edges [24].

In this paper, we investigated the movement of herbivore, natural enemy, pollinator and detritivore insects at a forest-crop interface, simultaneously considering for the first time the effects of forest amount in the landscape and crop phenology, while also assessing forest contribution to insect communities in nearby cultivated fields. We emphasize the focus on herbivores and natural enemies, due to their relevance in agroecosystems as possible pests and biological control agents, respectively. By placing bi-directional flight interception traps at the boundary between soybean fields and remnants of Chaco Serrano forest, in nine circular landscapes with a gradient of forest cover and at three soybean phenological stages (vegetative, reproductive and senescent), we aimed to answer three questions: (1) Is the magnitude (number of species and individuals) and direction of insect movement at the forest-crop boundary influenced by soybean phenological stage and forest cover in the landscape? We addressed this question for each of the above mentioned functional groups and also for their major orders, so as to assess possible effects of differential flying behavior on trap captures [25]; (2) Do herbivore and natural enemy communities moving from forest to soybean differ from those moving in the opposite direction, and how is their composition influenced by crop phenology and forest cover?; (3) Does movement from the forest to the crop represent a “spillover” [1] that influences the composition of insect communities within soybean fields?

We expected (1) movement intensity and direction to change along the crop cycle, with more insects moving from forest to crop during vegetative and flowering phases, while switching to a predominant crop-to-forest movement during crop senescence. However, this general trend could vary among functional groups. For herbivores, an early response to crop resources (high movement during vegetative stage) was expected. Natural enemies would show a delayed response [26], increasing their forest to crop movement during flowering, following their resource populations and taking advantage of additional resources like nectar and pollen in the crop. On the other hand, movement of pollinators would increase in both directions at the time of crop flowering, which would promote interchange between nesting and foraging habitats. Moreover, since landscapes with larger amounts of natural habitats usually sustain higher richness and/or abundance of insects, we expected movement intensity to be positively related with forest cover. Independently of changes in total number of species or individuals, particular species may show a directional movement either towards the crop or towards the forest [27], therefore we expected (2) low similarity in the communities moving in each direction, particularly at the beginning and end of the season when movement should be more directional. Finally, if insect movement from the forest to the crop indicates forest contribution to the makeup of soybean insect communities, we expected (3) similarity between assemblages moving towards soybean and those found within soybean fields to increase with forest proximity (because of insect flight limitations) and with forest cover (because of enhanced insect interchange).

## Methods

### Study area and sampling design

Sampling was performed from December 2010 to March 2011 at Córdoba province, in central Argentina (31.10°–31.30°S and 64°–64.30°W). The area belongs to the Chaco Serrano phytogeographical district, with 750 mm annual rainfall and average monthly temperatures between 26°C (maximum) and 10°C (minimum) [28]. Over the last 30 years, 94% of the Chaco Serrano was lost [29] in a process closely related to the expansion of croplands [30]. Soybean (*Glycine max* L.) has recently become the main crop in Argentina, covering over 15 million ha and leading to the loss of native habitats and displacement of other crops [31]. Using the Landsat Thematic Mapper and field corroboration, nine landscape circles of 500 m diameter were selected (hereafter referred to as sites), encompassing a gradient of forest cover proportion from 0.05 to 0.79, the remaining surface being occupied by soybean fields. This scale has effectively explained biodiversity and ecosystem processes variations in previous studies [32–34] and it insured independence of sample sites in our study. Landscapes were separated on average by 1.2 km (±0.5) and the woodland remnants have been isolated for at least 40 years.

### Ethics statement

No specific permits were required for the field studies here reported. Samplings were performed at Estancia Santo Domingo, whose owners authorize researchers of the Universidad Nacional de Córdoba to access forest and crops within their land.

### Arthropod sampling

At each site, one bi-directional flight interception trap (also known as window trap) [35] was placed at and parallel to the forest edge—soybean limit in order to assess movement between the two adjacent habitats. Only forest edges facing west were selected, in order to standardize potential wind effects. Traps had a surface capture of 0.6 m^2^ (100 x 60 cm) provided by a transparent PVC sheet stretched between two standing iron rods and placed 50 cm above the ground. Insects were collected in a plastic trough (11 cm diameter) below each side of the sheet, half-filled with 20% ethylene glycol. Bi-directional traps allow the study of insect movement direction, since insects flying perpendicular to the trap will hit the capture surface and fall into the trough. However, they have rarely been used to analyze temporal changes in the crop/non-crop boundary [20,36]. It is assumed that insects are intercepted randomly by these traps [35] and even if a few groups might avoid them [20,35] this would not affect the directionality comparison.

Traps were active at three one-week periods, which coincided with specific phases of soybean growth cycle: vegetative (13-20/12/2010), reproductive (20-27/01/2011) and crop senescence (28/02-06/03/2011). At the end of each period, the contents of each side of the traps were filtered, placed in plastic cups with 70% ethanol (labeled with site, movement direction and sampling period) and taken to the laboratory. All captured arthropods were identified to family level and assigned to functional groups on the basis of family habits (or subfamily, for families with multiple feeding habits) [37]. Natural enemies (predators and parasitoids), herbivores, pollinators and detritivores were then classified to morphospecies (further referred to as species) [38] and considered for further analyses. Lepidoptera specimens were assigned to the herbivore group due to their relevance as soybean pests [39] and because they are not considered important pollinators for this crop [40].

In order to compare insects moving between forest and crop (i.e. captured with flight interception traps) with insect communities in the soybean field, yellow pan traps [41] were placed in the crop at 5, 25, 50 and 100 m from the forest. These traps (diameter 34 cm, depth 9 cm) were placed at ground level, filled with 3 liters of water to which a few drops of detergent were added, and were active for 3 days during the vegetative period (20–22/12/2010) almost at the same time as flight interception traps (for more details see [16]). After a general sorting at family level, herbivores and natural enemies in the pan trap catches were classified to morphospecies level, for comparisons with flight interception traps data.

### Statistical analysis

To analyze spatial (forest cover) and temporal (crop phenology) variations in the magnitude (= intensity) and direction of insect movement, we calculated abundance and species richness of the four functional groups captured with the flight interception traps and used them as response variables in Generalized Linear Mixed Models (GLMMs). Additional models were performed on abundance and richness of the dominant orders (those representing at least 5% of total abundance and richness) in each functional group. Poisson error distribution and log link function were used for richness, while a negative binomial error distribution was used to deal with overdispersion in abundance data. Forest cover proportion at each site (as a continuous variable), movement direction (as a factor with two levels: forest to crop, crop to forest) and crop phenology (as a factor with three levels corresponding to soybean phases: vegetative, reproductive, senescent) were the explanatory variables, and we included each pair of interactions between these variables (see expectations in Introduction section). The triple interaction was not included since we had no biological hypothesis associated to it. We also included site as a random factor to model data dependence within each landscape circle and because of the repeated measures nature of the study design. Analyses were performed using the software R (version 2.15.1) [42] and the package lme4 [43]. Model comparisons were made with AICc values (Akkaike information criterion with correction for finite sample size) [44] using the package MuMIn [45], and autocorrelation was checked using variograms of the residuals [46]. We compared the AICc values of all possible models, from the full model with all interactions to the null model, and selected the model with the lowest value of AICc as the top model. When differences in AICc were ≤2, we further compared the models by likelihood ratio tests [46].

In order to investigate to which extent species moving from the forest to the crop were also found moving in the opposite direction in our flight interception traps captures, we analyzed similarity between assemblages collected at each side within each trap. Similarity was calculated for every site and soybean phase using Chao Jaccard abundance-based estimator [47]. Bias related to sample size is reduced in this index by estimating the probability that individuals belong to shared species and accounting for the effect of unseen shared species [47]. Chao Jaccard estimator was calculated using EstimateS (version 9.1.0) [48]. Then, using this index as response variable, we performed a GLMM with forest cover, crop phenology and their interaction as independent variables, with a normal error distribution and using the nlme package [49].

Finally, forest contribution via spillover to within-crop herbivore and natural enemy communities was assessed by analyzing the similitude between flight interception and pan trap samples. Thus we calculated, for each site, the Chao Jaccard Index between flight interception traps samples with forest to crop direction and yellow pan trap samples at each of the four distances to the forest (5, 25, 50 and 100m). Then, we ran GLMMs with Chao Jaccard Index as response variable against forest cover, distance to the forest and cover x distance interaction as independent variables. Site was also included as random factor and model selection was carried out using AICc values as explained above.

## Results

### Magnitude of the movement between forest and soybean

**Fig 1:**
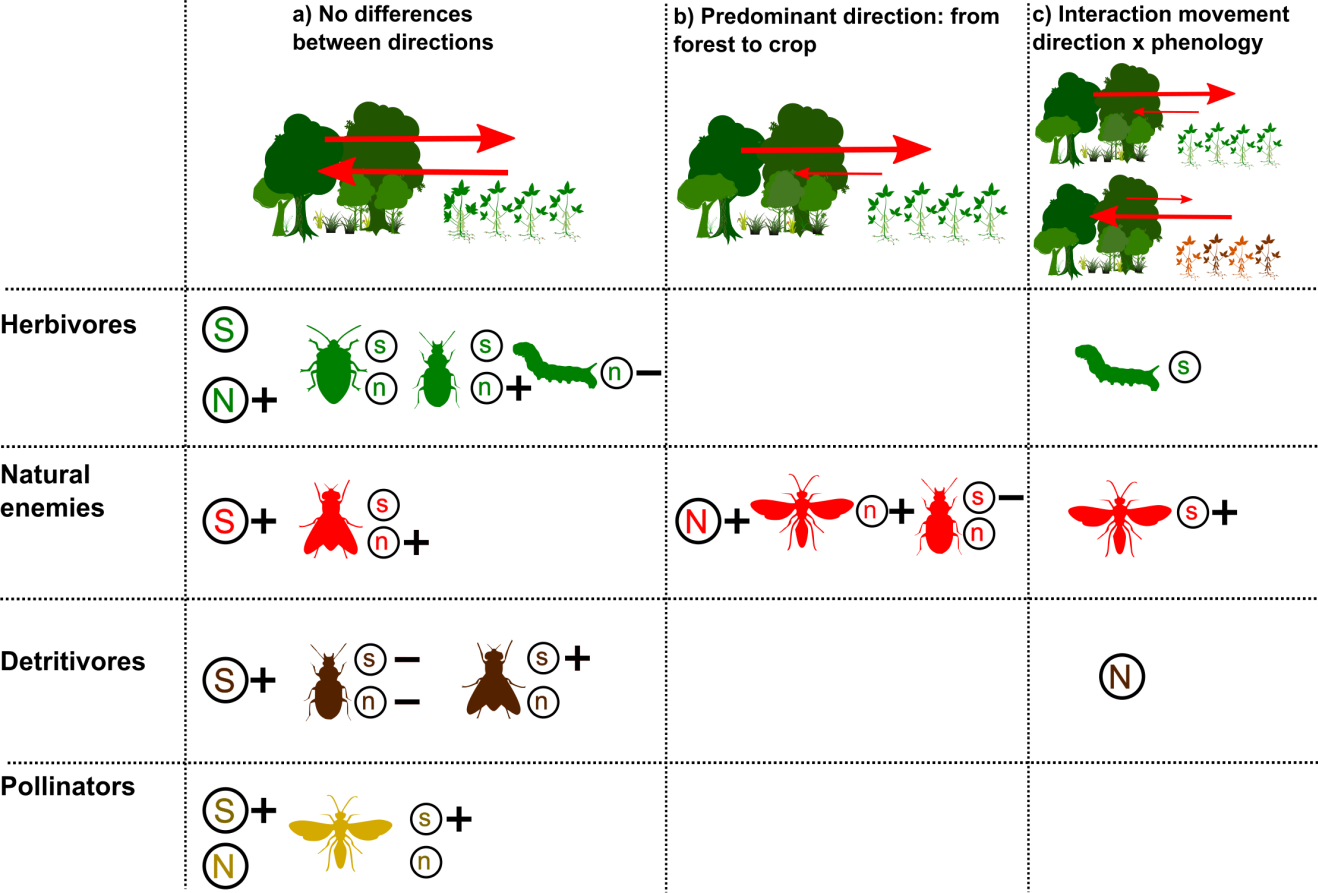
Main trends in insect interchange between Chaco Serrano forest and soybean crops. Summary of trends in insect movement intensity (as richness and abundance) at the forest-crop interface, considering movement direction and the effects of soybean phenology and forest cover. Responses were classified in three main types: (a) No significant differences between movement directions at any of the soybean phases; (b) More intense movement from the forest to the crop throughout the crop cycle; and (c) Interaction between movement direction and soybean phenology, when the predominant movement direction changed between phases. Insect orders are shown as representative silhouettes, with color indicating functional group (herbivores: green, natural enemies: red, detritivores: brown, pollinators: yellow). Abundance (N) and richness (S) are represented in capitals for the responses of total functional groups, while lowercase and representative drawings are used for the dominant orders of each group. Relationships between movement intensity and forest cover are indicated with + for positive and–for negative relationships when they were detected in at least one of the crop phases (see Table 1 for details of each case).

**Fig 2:**
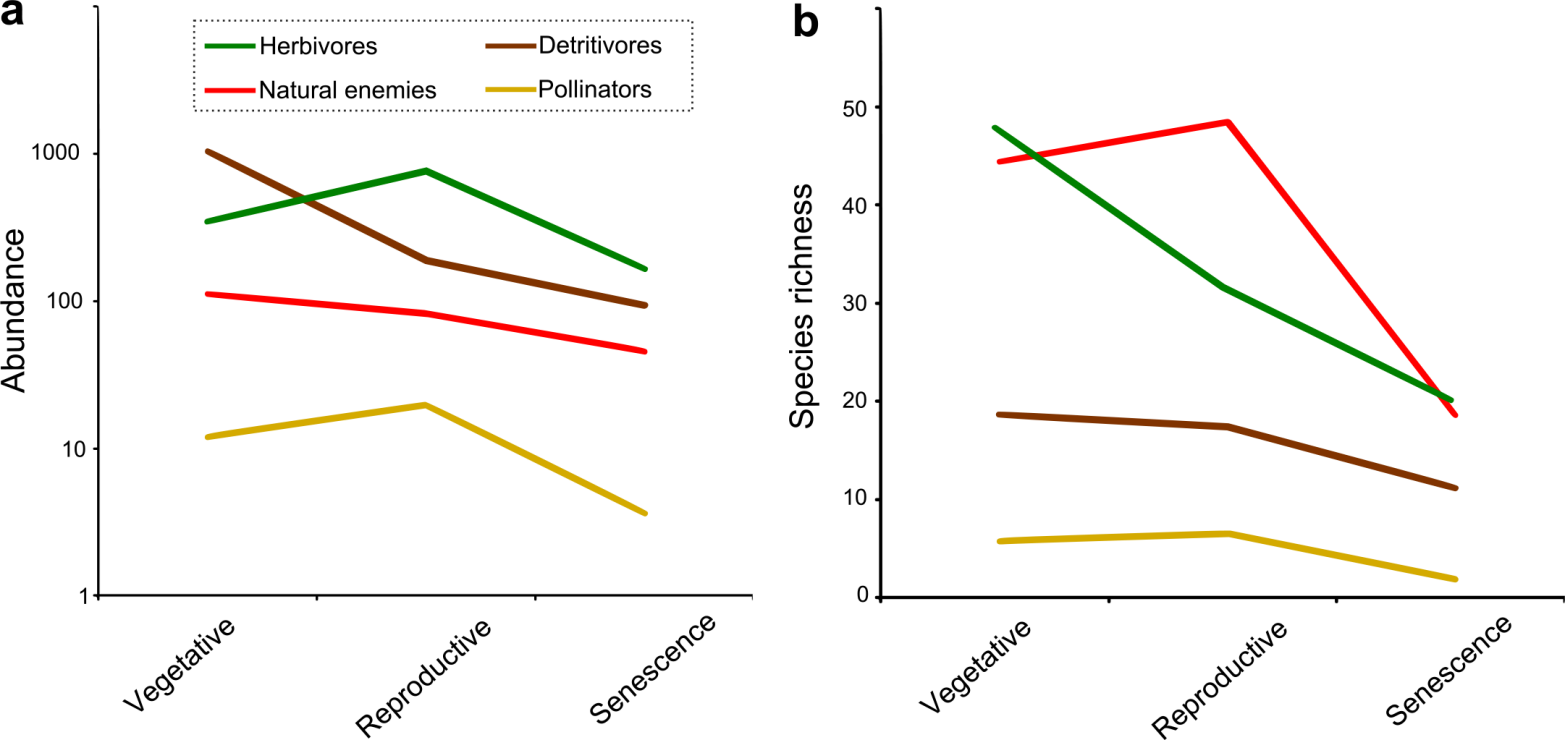
Temporal variations in movement intensity of insect functional groups. Mean richness (a, left) and abundance (b, right) at each of the three soybean phases for the functional groups of insects collected with flight interception traps: herbivores (green), natural enemies (red), detritivores (brown) and pollinators (yellow).

Our flight interception traps captured 52,173 arthropods belonging to 877 species. Up to 5,450 individuals and 255 species per trap were collected within a sampling week. Herbivores were well represented, with 44% of all specimens and 30% of the species collected, among which Coleoptera, Hemiptera and Lepidoptera were the most diverse orders. Natural enemies, although less abundant (8% of individuals) were the most diverse group (51% of captured species), with Coleoptera, Diptera and Hymenoptera being the most representative orders. Pollinators were dominated by Hymenoptera and poorly represented (2% of specimens and 7% of species), while detritivores, best represented by Coleoptera and Diptera, were most abundant (46% of total individuals) but contributed just 8% of the total diversity. Fig 1 summarizes trends in movement direction with regard to forest cover and crop phenology for these functional groups and their main orders, while Fig 2 shows their temporal variations in richness and abundance.

According to the model best fitting our data (S1 Table) the number of herbivore species moving between forest and soybean did not vary between directions or with forest cover (Fig 1A), but decreased along the soybean growing season (Table 1; Fig 2; S1A Fig). Herbivore abundance did not differ between directions either, but was highest at soybean reproductive phase, lowest at senescence (Fig 2), and increased with forest cover at both these periods (interaction between crop phenology and forest cover) (Table 1; S1B and S2A Figs). Within the dominant herbivore orders, different patterns emerged. For Hemiptera, both richness and abundance showed only temporal variations, decreasing as crop phenology advanced (Table 1; S1 Fig). Coleoptera richness and abundance decreased along the soybean season (S1 Fig) and (marginally) more specimens were captured at sites with larger forest cover (Table 1; S2B Fig). Finally, more Lepidoptera species moved from forest to soybean during the vegetative phase, reverting this tendency at later periods (significant movement direction x crop phenology interaction) (Table 1; S1A Fig), whereas more specimens of Lepidoptera were also captured during the vegetative phase and in sites with reduced forest cover (significant forest cover x crop phenology interaction) (Table 1; S2C Fig).

**Table 1:** Analyses of forest-crop movement intensity for insect functional groups and their dominant orders.

Functional group	Order	Response variable	Movement direction	Forest cover	Crop phenology	Direction x Forest cover		Direction x Phenology	Forest cover x Phenology
Herbivores	Total	Richness^nb^	-	-	**<0.001**		-	-	-
		Abundance^nb^	-	0.64	**0.0006**		-	-	**0.03**
	Coleoptera	Richness^p^	-	-	**<0.001**		-	-	-
		Abundance^nb^	-	0.09	**<0.001**		-	-	-
	Hemiptera	Richness^p^	-	-	**<0.001**		-	-	-
		Abundance^nb^	-	-	**<0.001**		-	-	-
	Lepidoptera	Richness^p^	0.42	-	**<0.001**		-	**0.05**	-
		Abundance^nb^	-	**0.03**	**<0.001**		-	-	**0.003**
Natural enemies	Total	Richness^nb^	-	0.47	**<0.001**		-	-	**0.008**
		Abundance^nb^	0.07	0.91	**<0.001**		-	-	**0.02**
	Coleoptera	Richness^p^	**0.002**	**0.03**	**<0.001**		-	-	-
		Abundance^nb^	**0.01**	-	**<0.001**		-	-	-
	Diptera	Richness^p^	-	0.09	**<0.001**		-	-	**0.02**
		Abundance^nb^	-	-	**<0.001**		-	-	-
	Hymenoptera	Richness^nb^	**0.009**	0.21	**<0.001**		**0.008**	**0.001**	-
		Abundance^nb^	0.09	-	**<0.001**		-	-	**0.001**
Detritivores	Total	Richness^nb^	-	0.18	**<0.001**		**0.02**	-	-
		Abundance^nb^	0.43	-	**<0.001**		-	**0.004**	-
	Coleoptera	Richness^p^	-	**0.004**	**<0.001**		-	-	**0.009**
		Abundance^nb^	-	**0.03**	**<0.001**		-	-	**0.02**
	Diptera	Richness^p^	-	**0.007**	**<0.001**		-	-	-
		Abundance^nb^	-	-	**<0.001**		-	-	-
Pollinators	Total	Richness^p^	-	0.71	**<0.001**		-	-	**0.002**
		Abundance^nb^	-	-	**<0.001**		-	-	-
	Hymenoptera	Richness^p^		0.94	**<0.001**		-	-	**0.009**
		Abundance^nb^	-	-	**<0.001**		-	-	-

Results of GLMM analyses of insect movement (as numbers of species and specimens captured in bi-directional flight interception traps) between Chaco Serrano forest and soybean crops. Models included movement direction, proportion of forest cover in the landscape, crop phenology and the interactions between these variables. The superscript in each response variable indicates the error distribution used in the model (nb = negative binomial; p = Poisson). Only P-values of explanatory variables in the best model for each response variable are included (in bold, P-values ≤0.05).

Natural enemies also showed similar number of species moving in both directions (Fig 1A) and a significant reduction along the crop cycle (Fig 2), but differed from herbivores by increasing their diversity with the amount of forest, during crop senescence (forest cover x crop phenology interaction) (Table 1; S3A and S4A Figs). Natural enemy abundance followed the same trends, showing in addition a marginally predominant movement from the forest to the crop all along the season (Table 1; Fig 1B; S3B and S4B Figs). Within the dominant natural enemy orders, the number of Diptera species decreased at senescence, but increased with forest cover at the vegetative and senescence phases (significant crop phenology x forest cover interaction) (Table 1; S3A and S4C Figs); their abundance showed only temporal variations, being highest during soybean vegetative phase (Table 1; S3B Fig). At all times, more species and specimens of Coleopteran natural enemies moved from forest to soybean than in the opposite direction (Fig 1B); moreover both richness and abundance decreased at senescence (S3 Fig), with fewer species being captured in landscapes with higher forest cover (Table 1; S4D Fig). For Hymenoptera, more species moved from the forest to the crop at vegetative phase, balancing numbers in both directions at reproductive phase and finally reversing to a predominant crop to forest movement at senescence, when fewest species where captured (direction x crop phenology interaction) (Table 1; Fig 1C; S3A Fig). There was, in addition, an interaction between movement direction and forest cover, since the number of species moving from the crop to the forest was positively related with forest amount in the landscape (S4E Fig). More individuals of Hymenoptera moved from the forest to the crop all along the season (Table 1; Fig 1B; S3B Fig) and, only at crop senescence when they were rarer, their abundance increased with forest cover (significant crop phenology x forest cover interaction; S4F Fig).

There was no difference in the numbers of pollinator species moving in each direction (Fig 1A), but they increased with forest cover at soybean senescence, when fewer species were observed (crop phenology x forest cover interaction) (Table 1; S5A and S6A Figs). Pollinator abundance was, as expected, higher at soybean flowering and lower at senescence, with no effects of forest cover or movement direction (Table 1; S5B Fig). Hymenoptera, encompassing 75% of the specimens and 79% of pollinator species, repeated the group trends: a significant forest cover x crop phenology interaction for richness (Table 1; S6B Fig) and only temporal changes in abundance i.e. more individuals moving during crop flowering (S5B Fig).

The species richness of detritivores was lowest at senescence (Fig 2) and showed an interaction between movement direction and forest cover: while the number of species moving from the forest to the crop was independent of forest cover, more species moved from soybean to forest as forest cover increased (Table 1; S8A Fig). The abundance of detritivorous insects was also lowest at senescence (Fig 2), and then more individuals moved from the crop to the forest than on the opposite direction (movement direction x crop phenology interaction; Fig 1C). There were no differences between directions at other phases, but abundance was remarkably higher at vegetative phase (Table 1; S7B Fig). Coloptera and Diptera were the best represented taxa within this functional group. Neither abundance nor richness of coleopteran detritivores differed between directions but both variables were negatively related to forest cover, albeit only at the vegetative phase (forest cover x crop phenology interaction) (Table 1; S8B and S8C Fig). Richness and abundance of Diptera were also similar between both movement directions and decreased along the soybean cycle (Table 1; S7 Fig), with more species at sites with larger forest cover (S8D Fig).

### Composition of communities moving in each direction

**Fig 3:**
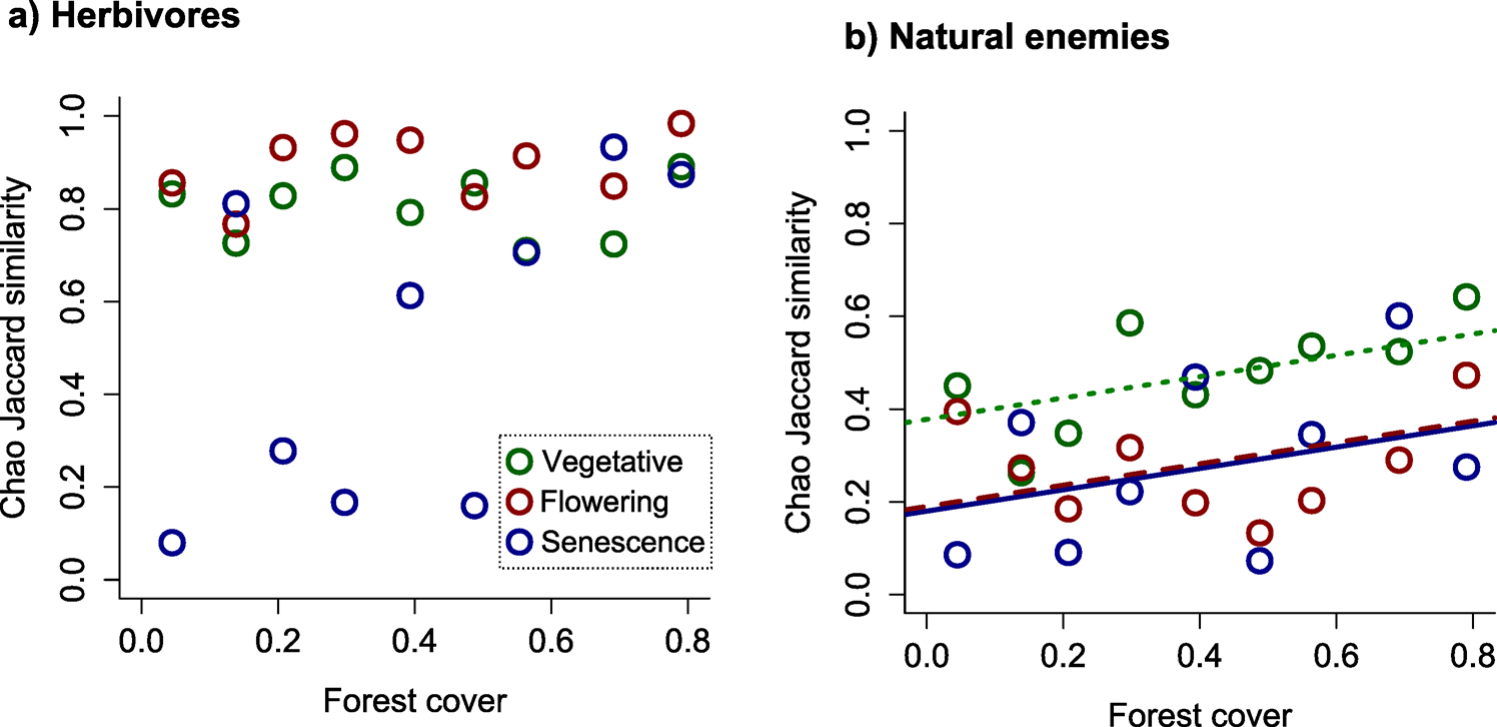
Similarity in communities moving in either direction between Chaco Serrano forest and soybean. Within-trap similarity in community composition for (a) herbivores and (b) natural enemies captured at each side of flight interception traps, in relation to forest cover and soybean phases.

When the taxonomic composition of communities captured at either side of the flight interception traps was compared, in order to assess whether the same or different species were flying in each direction, herbivores presented a high level of similarity (0.73 ± 0.05 mean value of Chao-Jaccard estimator along the three phases) indicating that species moved indistinctly in both directions. The degree of similarity between herbivore communities flying to the crop and those moving to the forest was independent of forest cover but was influenced by crop phenology, presenting the lowest values at senescence (Table 2; Fig 3A). For natural enemies, the overall similarity between communities moving to and from the forest represented less than half the values observed for herbivores (0.34 ± 0.03). Their Chao-Jaccard values were higher during the vegetative phase and increased with forest cover all along the crop cycle (Table 2; Fig 3B).

**Table 2:** Similarity in composition of communities moving in either direction at the forest-crop boundary.

Functional group	Forest cover	Crop phenology	Interaction
Herbivores	0.48	**0.0006**	0.17
Natural enemies	**0.05**	**0.007**	0.49

Results of GLMM analyses of within-trap similarity in community composition for herbivores and natural enemies captured at each side of flight interception traps, in relation to forest cover and crop phenology. Significant effects (P-values ≤0.05) are indicated in bold.

### Forest spillover influence on soybean insect communities

**Fig 4:**
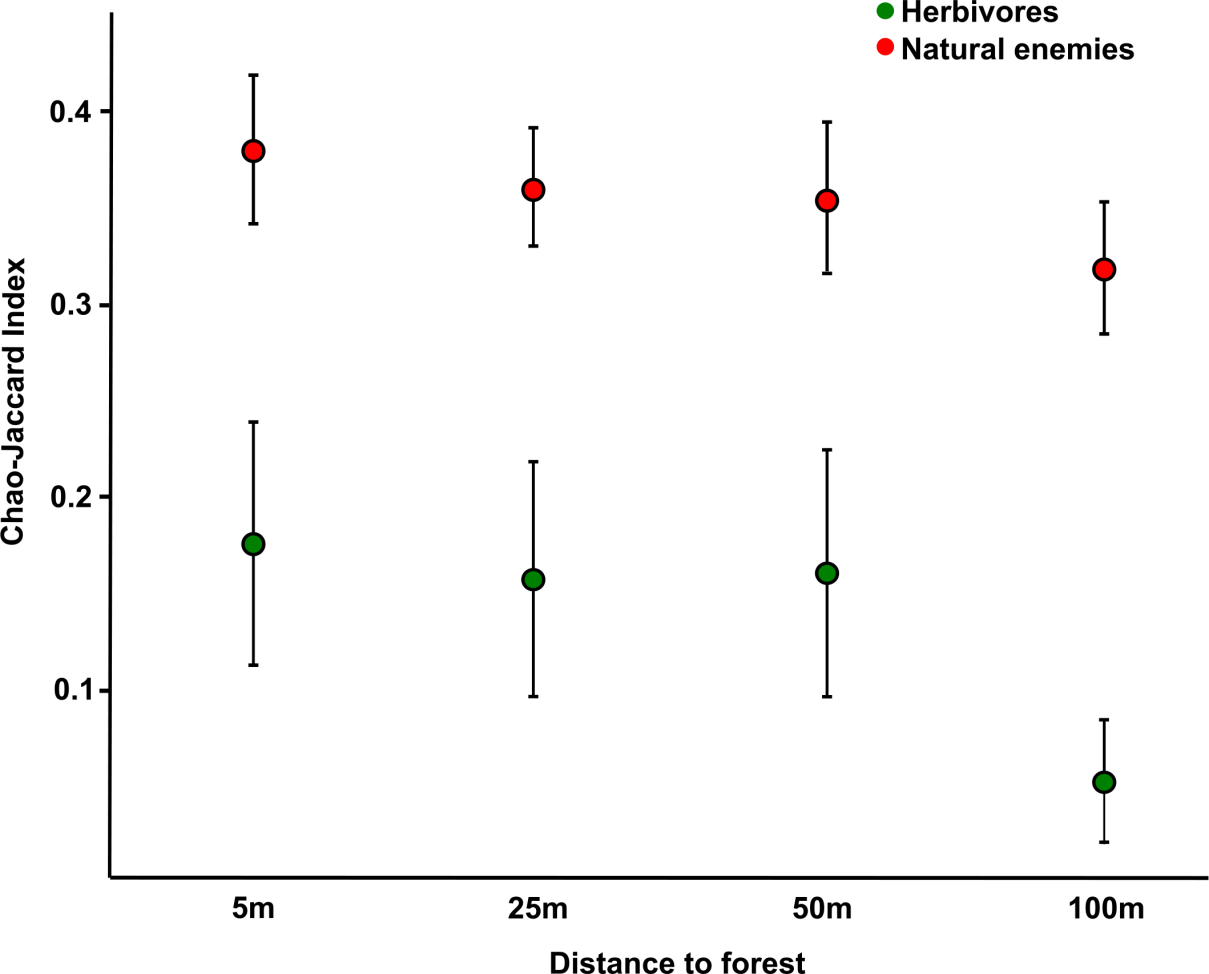
Influence of forest-to-crop movement on soybean assemblages. Similarity in community composition between insects captured at the forest- to- crop side of flight interception traps and those captured with yellow pan traps placed on soybean at 5, 25, 50 and 100 meters from forest edge. Herbivores are represented in green and natural enemies in red.

Herbivores captured within soybean fields using yellow pan traps presented a mean similarity of 0.14 ± 0.05 with communities moving from the forest to the crop in flight interception traps captures. The Chao-Jaccard index values were not related to forest cover, but showed slight differences at local scale, with lower similarity values at 100m from the forest in comparison with shorter distances (Table 3; Fig 4). For natural enemies, mean similarity between communities moving from the forest and communities captured on soybean was twice the value estimated for herbivores (0.36 ± 0.04). Again, forest cover did not affect this variable, but a slight change was observed at local scale: communities captured at 5m from the forest presented higher similarity between trap types than those captured at 100m, with intermediate values at 25 and 50m (Table 3; Fig 4).

**Table 3:** Influence of movement from the forest on to community composition of crop assemblages in the crop.

Functional group	Forest cover	Distance to forest	Interaction
Herbivores	0.39	**0.04**	0.98
Natural enemies	0.76	**0.03**	0.18

Results of GLMM analyzing similarity between communities moving from the forest to the crop and those captured within soybean fields, for herbivores and natural enemies. Forest cover in the landscape, distance to forest and the interaction between these variables were evaluated, with significant values (P-values ≤0.05) in bold.

## Discussion

The interchange of insects between natural and cultivated habitats is acknowledged as a common process underlying richness and abundance patterns in both types of ecosystems [12]. However, despite a variety of methods designed to record directional insect movement [50] and a large body of literature about the influence of natural environments on the biodiversity of agroecosystems [15,18], surprisingly few studies have focused on the directionality of insect movement between adjacent crop and non-crop habitats (ie. [20,36,51,52]). In this paper, we offer the first simultaneous analysis of the effects of forest amount in the landscape and crop phenology on insect movement at a forest-crop interface, while also assessing forest contribution to insect communities in nearby cultivated fields. We found that while movement patterns varied among functional and taxonomic groups, a predominantly forest-to-crop movement was remarkably restricted to natural enemies (Fig 1); we also found the intensity of insect movement to be extensively affected by crop phenology and mostly enhanced by forest cover in the landscape (Table 1). In addition, we showed that mainly the same herbivore species moved to and from the forest, whereas natural enemy species displayed a more directional movement (Fig 3). Finally, our analyses revealed forest contribution to insect communities in the crop to be greater for natural enemies than for herbivores, fading with distance to the forest in both groups (Fig 4).

### Magnitude and direction of insect movement vs crop phenology

Annual crops like soybean are periodically subjected to mechanical [53] and chemical [54] disturbances so they must be regularly colonized by insects from adjacent crops or natural habitats [55]. Immigration to crops is expected to occur mainly after sowing, reversing at the time of crop senescence or harvest, when insects seek refuges in non-crop habitats [1]. With more than 5,000 individuals in over 250 species flying within one week across an imaginary 0.6 m^2^ window, our interception trap captures revealed an intense insect interchange between forest and soybean fields, mostly with similar numbers of species and/or individuals moving in either direction and generally declining as the crop entered senescence (Figs 1 and 2; Table 1). Thus our overall results suggest daily or periodic displacements, with insects moving to and from and using both habitats within hours or days, a trend more akin to a continuous process throughout the crop cycle [20] than to our expectation of forests acting alternatively as source and sink of insects depending on crop phenology. The fading intensity of insect interchange during crop senescence could be linked to the sharpened edge contrast at that stage, since such hard edges are usually characterized by low permeability and limited interchange [2]. For example, Collinge & Palmer [3] found that ground-dwelling beetles were less likely to move across habitats when patches were embedded within a high contrast matrix, as would be in the case of senescent soybean when marked changes in plant pigmentation, resource quality and availability and sunlight exposition would increase contrast with the forest.

Studies on cross-edge spillover of insects between anthropogenic and natural habitats usually consider a directional movement i.e. insects dispersing actively in a given direction [1], however the interchange between crop and non-crop habitats can also be the result of routine or trivial movements [56] which seems a plausible explanation for our results. Insect interchange, even through trivial displacements, may have important consequences for ecosystem processes in the forest and in the crop [17], especially because organisms found at the interface are the most likely to cross habitat boundaries [5]. In pollinators, for example, the lack of differences between captures at either side of our bidirectional traps, could reflect periodic flights between forest and crop or daily activity patterns [57], in order to use crop resources while satisfying their need for natural vegetation to complete their cycles [17].

In an important exception to the general trend described above, natural enemies showed various instances of directional movement (Fig 1; S3 Fig). Overall, more natural enemies (particularly in Coleoptera and Hymenoptera) moved from the forest toward the crop than viceversa. Given that natural enemies were the most diverse group moving in the forest-soybean interface, and considering previous observations of increased natural enemy richness in the forest proximity [16], these results suggest an interesting role for Chaco Serrano remnants as sources of natural enemies for soybean pests. Conversely, at the end of the crop cycle the forest may become a refuge from the barren matrix, as suggested by Hymenopteran natural enemies returning to the remnants of Chaco Serrano during soybean senescence.

Only two other instances of differential movement directions interacting with crop phenology were observed. One of them was provided by detritivores moving predominantly toward the forest at senescence, after moving indistinctly in both directions during earlier crop phases (S7 Fig). This was unexpected since no-tillage farming was used in the study sites, a system favoring the development of insect populations and resources for detritivores [58,59], however the need of additional resources or refuges provided by the forest appeared to be still relevant for this group. The other case was represented by Lepidoptera within herbivores, with more species moving from forest to soybean than in the opposite direction at the vegetative phase, and reverting to the forest during flowering and senescence (S1 Fig). These changes could be explained by variations in resource availability, since various defoliating Lepidoptera are well known pests in early stages of soybean phenology [39], when adults could be expected to fly from the forest to lay their eggs on the crop. Although soybean still offers resources for defoliating larvae at flowering [60], changes in plant quality or intra and interspecific competition due to high herbivore density, could promote relatively early movement away from the crop, since these conditions can lead to dispersal [5] and emigration [61].

### Forest cover and insect interchange

The amount of non-crop habitats in the landscape is known to enhance insect diversity, ecological processes and ecosystem services in crops [15,62], most likely via increased inter-habitat movement of organisms when larger natural habitats are present, as suggested by a simulation study [63]. However, the relationship between insect interchange and amount of non-crop habitats has been generally ignored. This first exploration of the issue revealed a predominantly positive link between the amount of forest cover in the landscape and the intensity of insect movement at the forest-crop interface, for the functional groups here analyzed (Fig 1; Table 1).

Thus, as forest cover in the landscape decreased, fewer species of natural enemies, detritivores and pollinators were found moving between forest and crop (S4, S6 and S8 Figs). The enhancement of insect interchange in landscapes with higher proportion of forest cover becomes particularly relevant in the light of previous findings from the same region, which showed ecological processes such as parasitism [64,65] and litter decomposition [66] in Chaco Serrano to improve with increasing forest size. At least for parasitism, such improvement was strongly linked to increased insect diversity [67]. Consequently, a richer forest-crop interchange may be expected to boost ecosystem services such as biological control, nutrient recycling or pollination in landscapes with relatively large forest cover.

The relationship between the number of insect species involved in crop / non-crop interchange and the amount of forest in the landscape was observed mostly during crop senescence when insect movement was generally lower. Insect interchange dropped noticeably during senescence in sites with lower forest cover but kept relatively homogeneous levels along the season in forest-dominated landscapes. Small remnants of natural vegetation, which are predominant in simplified landscapes [68] like our crop-dominated sites, tend to provide only temporal or semi-permanent habitats [69] and consequently, their role as sources of insects for crop / non-crop interchange would be limited in time. Therefore, by favoring temporal stability of insect movement, the larger forest cover found in our complex landscapes could lead to higher resilience in ecosystem services [70] involving natural enemies, pollinators or detritivores.

Increased forest cover also resulted in intensified herbivore exchange, along the flowering and senescence stages (S2 Fig). Given that forest-dominated landscapes offer fewer resources for crop herbivores [71], competition and resource limitation could turn the relatively scarce cultivated area into a low quality habitat, which would be assesed by the insects, increasing the probability of movement in search of a better habitat [5,72].

Contrary to the general positive pattern described above, a few insect groups responded negatively to forest cover. Fewer Lepidoptera moved across the edge at higher forest cover during the vegetative phase (S2C Fig). Since various species of Lepidoptera are important soybean pests, their diminished interchange in landscapes with a high proportion of natural habitats may mean a reduced provision of ecosystem dis-services [6] from the forest to soybean fields. On the other hand, the intense movement in simplified landscapes could have negative consequences for native vegetation, such as damage to leaves and fruits [73] or reduction in seed set [74], given that small fragments tend to be particularly vulnerable to herbivore spillover from crops [17]. A negative influence of forest cover on insect interchange was also observed for predatory and detritivorous species in Coleoptera (S4D and S8B Figs). Increased predator movement in crop-dominated landscapes where this group tends to be impoverished [15] could have positive consequences for soybean, but the above mentioned negative spillover effects on non-pest herbivores in the forest are likely to be still more important [1].

### Composition of communities moving in each direction and spillover influence on soybean insects

Within herbivore communities, the species moving from the forest to the crop were frequently also found moving in the opposite direction, according to the high levels of similarity between captures at each side of each interception trap (Fig 3A; Table 2). In other words, not only the numbers of herbivore species and individuals moving to and from the forest were similar, but they were mostly the same species, supporting the idea of boundary crossing mainly as the result of trivial movements rather than directional flights toward either crops or forests [56].

On the other hand, natural enemy species appeared to be more distinctly associated to a particular movement direction than their prey or hosts, with remarkably lower similarity values (Fig 3B). Thus, even when the number of species moving in each direction was similar, the species themselves were different, i.e. some species moved more frequently toward the forest while others followed the opposite trend. Such directionality could result in a net positive contribution of the forest to the agricultural system, given that natural enemies flying toward soybean were at all times more abundant than those flying toward the forest, as reported above. Our results are in line with previous evidence suggesting that natural enemies are more likely to move between habitats [20] and to benefit from landscape complexity and the resources present in natural vegetation [15] in comparison with herbivores. Moreover, in forest-dominated landscapes we found a growing representation of natural enemy species moving indistinctly in both directions, as expected if forests offer more alternative resources in this type of landscapes [18] that are used periodically by predators and parasitoids. Furthermore, similarity between communities moving in one or other direction decreased along the soybean cycle, suggesting that scarce matrix resources during crop senescence may promote movement directionality.

In coincidence with their greater directionality, similarity between natural enemies moving from the forest to the crop and those captured on soybean duplicated the values observed in herbivores (Fig 4). In other words, the predominant forest to crop movement of natural enemies seems to determine a greater contribution to the structure of soybean communities in comparison with herbivores. Supporting this result, there is evidence of in-crop density of natural enemies showing a stronger response than herbivores to nearby forest presence in the landscape [75]. Moreover, such forest contribution to crop communities decreased at larger distances in both groups, as observed in other studies addressing changes in diversity or abundance with proximity to non-crop habitats [14,16,76,77]. Nevertheless, the fact that at 100 meters from the forest the influence of forest spillover was almost null for herbivores, while still representing a substantial input for natural enemies in soybean, suggests a positive contribution from natural habitats to agroecosystems that must be highlighted.

## Conclusions

Summing up, our study has shown that larger amounts of forest in the landscape enhance insect interchange in four functional groups, mostly by engaging a wider diversity of species. Given that in most groups and most of the time, forest-to-crop and crop-to-forest insect movement were similarly intense, the relative allocation to each land use at landscape level could thus have consequences for the functioning of both ecosystems. In addition, we found that larger remnants of natural vegetation could promote temporal stability in ecosystem services by attenuating the generalized decline of insect interchange during crop senescence. Thus, crops in landscapes with high forest cover would receive a continuous influx of insects and therefore present higher levels of pest control or pollination.

Natural enemies (especially Coleptera and Hymenoptera) deviated from the general non-directional trend by moving most frequently from the forest to the crop, probably due to particular species migrating to and remaining in the crop for longer periods than non-directional species. Such natural enemy spillover was also boosted by increased forest cover and showed a remarkably stronger influence on the composition of soybean communities in comparison with that of herbivores. Altogether, these results support the contention of natural enemies benefiting more than pests from natural vegetation [20] and suggest a likely contribution to pest control in the cultivated fields.

Insect interchange between crop and non-crop habitats could involve both positive and negative consequences for agricultural production [17]. Our study suggests a balance tilted to the positive side, in terms of natural vegetation boosting diversity of insects engaged in such interchange. This could lead to improved pollination and nutrient recycling in both systems and, particularly, enhanced natural pest control in agroecosystems. Such results seem to advocate for a shared land use framework [78], that is, maintaining non-crop areas in agricultural landscapes in order to allow the coexistence of conservation and agricultural production. Nonetheless, consequences for the natural system deserve further study, especially considering the acceleration of insect movement towards the forest observed in particular taxa at specific times in crop phenology.

## Supporting Information

S1 Fig Movement intensity of herbivores between forest and soybean crops. Richness (a) and abundance (b) of total herbivores and the three main orders (Coleoptera, Hemiptera and Lepidoptera) moving toward crops (in green) and towards forest (in blue) at phenological phases of soybean (vegetative, reproductive, senescence).
(TIF)

S2 Fig Relationships between forest cover and herbivore movement. Significant relationships between proportion of forest cover in the landscape and movement of herbivore insects at phenological phases of soybean: vegetative (green), reproductive (red) and senescence (blue). Tendency lines are used only when the relation with forest cover was significant. (a) Total herbivore abundance. (b) Abundance of Coleoptera. (c) Abundance of Lepidoptera.
(TIF)

S3 Fig Movement intensity of natural enemies between forest and soybean crops. Richness (a) and abundance (b) of total natural enemies and their three main orders (Coleoptera, Diptera and Hymenoptera) moving toward crops (in green) and towards forest (in blue) at soybean phenological phases: vegetative, reproductive and senescence.
(TIF)

S4 Fig Relationships between forest cover and natural enemy movement. Significant relationships between proportion of forest cover in the landscape and movement of natural enemies at soybean phenological phases: vegetative (green), reproductive (red) and senescence (blue). When differences between movement directions were significant, empty circles represent movement from the forest and filled circles from the crop. Lines are used only when the relation with forest cover was significant, with dashed lines for movement from the forest, solid lines for movement from the crop and dotted lines for both directions. (a) Total enemy richness. (b) Total enemy abundance. (c) Diptera richness. (d) Coleoptera richness. (e) Hymenoptera richness. (f) Hymenoptera abundance.
(TIF)

S5 Fig Movement intensity of pollinators between forest and soybean crops. Richness (a) and abundance (b) of total and hymenopteran pollinators moving towards crops (in green) and towards forest (in blue) at soybean phenological phases: vegetative, reproductive and senescence.
(TIF)

S6 Fig Relationships between forest cover and pollinator movement. Significant relationships between forest cover in the landscape and movement of pollinator insects at soybean phenological phases: vegetative (green), reproductive (red) and senescence (blue). Lines are used only when the relation with forest cover was significant. (a) Total pollinator richness. (b) Hymenoptera richness.
(TIF)

S7 Fig Movement intensity of detritivores between forest and soybean crops. Richness (a) and abundance (b) of total detritivores and the two main orders (Coleoptera, and Diptera) moving towards crops (in green) and towards forest (in blue) at soybean phenological phases: vegetative, reproductive and senescence.
(TIF)

S8 Fig Relationships between forest cover and detritivore movement. Significant relationships between forest cover in the landscape and movement of detritivore insects at soybean phenological phases: vegetative (green), reproductive (red) and senescence (blue). When differences between movement directions were significant, empty circles represent movement from the forest and filled circles from the crop. Lines are used only when the relation with forest cover was significant, with dashed lines for movement from the forest, solid lines for movement from the crop and dotted lines for both directions. (a) Total detritivore richness. (b) Coleoptera richness. (c) Coleoptera abundance. (d) Diptera richness.
(TIF)

S1 Table Summary of model selection by AICc. For each functional group and order, the best three models and the full model (which includes all independent variables and their paired interactions) are ind1icated. AICc values for every model and ΔAICc (difference in AICc between each model and the top model) are also included. x indicates interactions between two variables. Independent variables abbreviations: ForestCov = Forest Cover in the landscape; CropPhen = Soybean Crop Phenology; MovDir = Movement Direction.
(DOCX)
