# Supplementary figures and images for: A Moveable Feast: Insects Moving at the Forest-Crop Interface Are Affected by Crop Phenology and the Amount of Forest in the Landscape
Source: PLoS One. 2016 Jul 6;11(7):e0158836. doi: 10.1371/journal.pone.0158836 (PMC4934915; doi:10.1371/journal.pone.0158836)

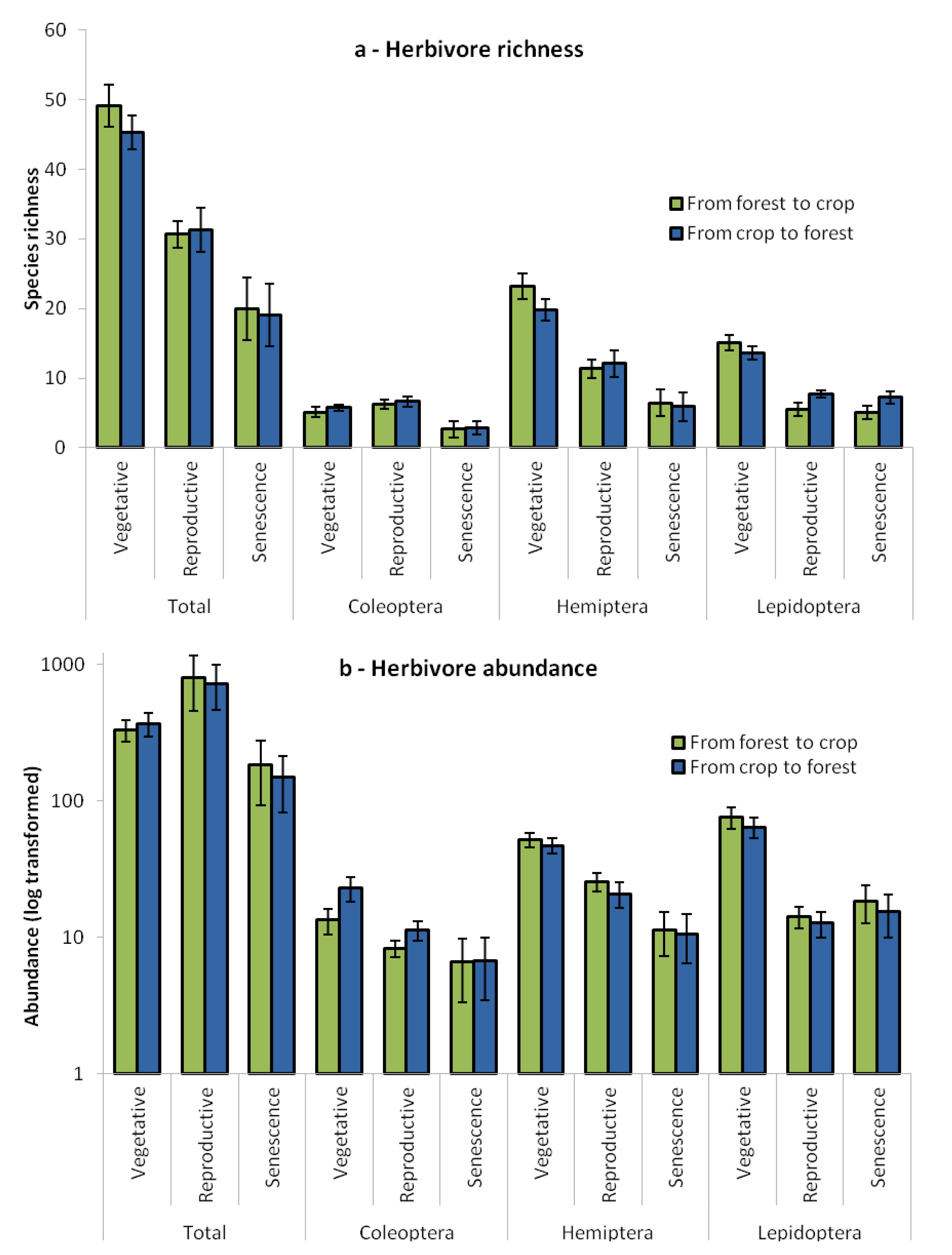

Supplement: S1 Fig — Richness (a) and abundance (b) of total herbivores and the three main orders (Coleoptera, Hemiptera and Lepidoptera) moving toward crops (in green) and towards forest (in blue) at phenological phases of soybean (vegetative, reproductive, senescence). (TIF) [file pone.0158836.s001.tif]

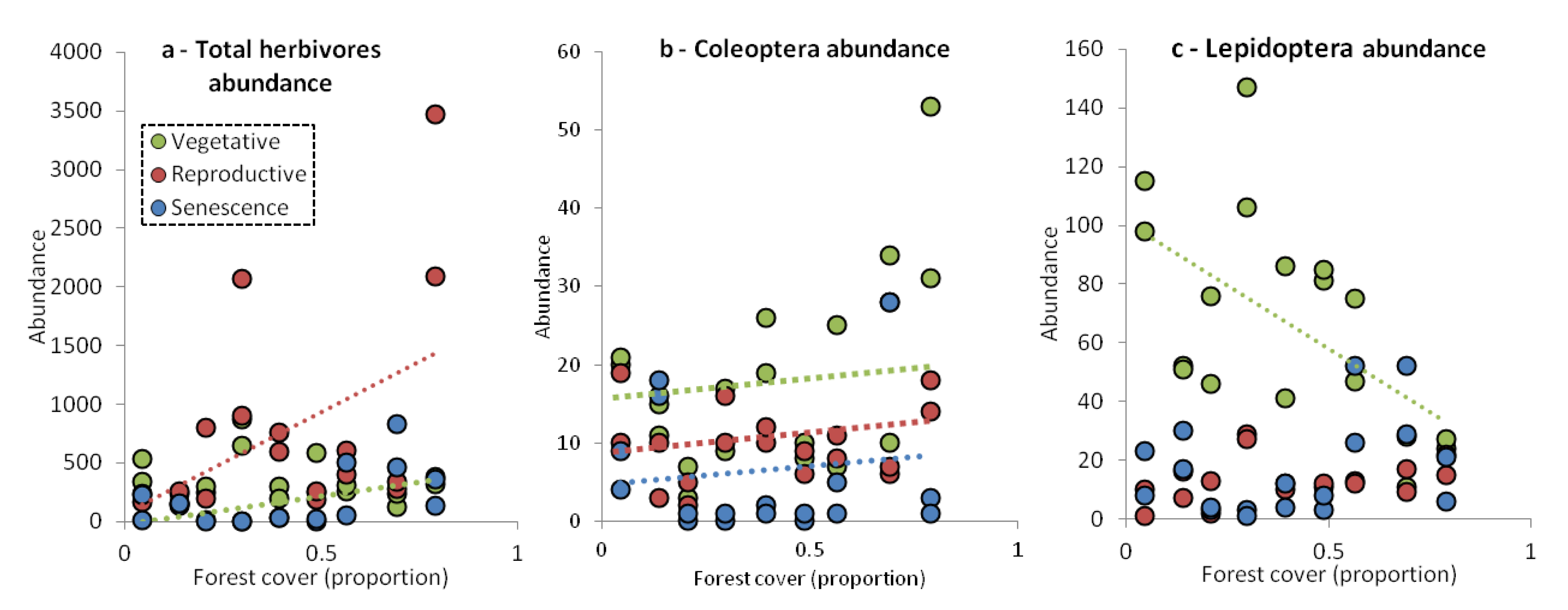

Supplement: S2 Fig — Significant relationships between proportion of forest cover in the landscape and movement of herbivore insects at phenological phases of soybean: vegetative (green), reproductive (red) and senescence (blue). Tendency lines are used only when the relation with forest cover was significant. (a) Total herbivore abundance. (b) Abundance of Coleoptera. (c) Abundance of Lepidoptera. (TIF) [file pone.0158836.s002.tif]

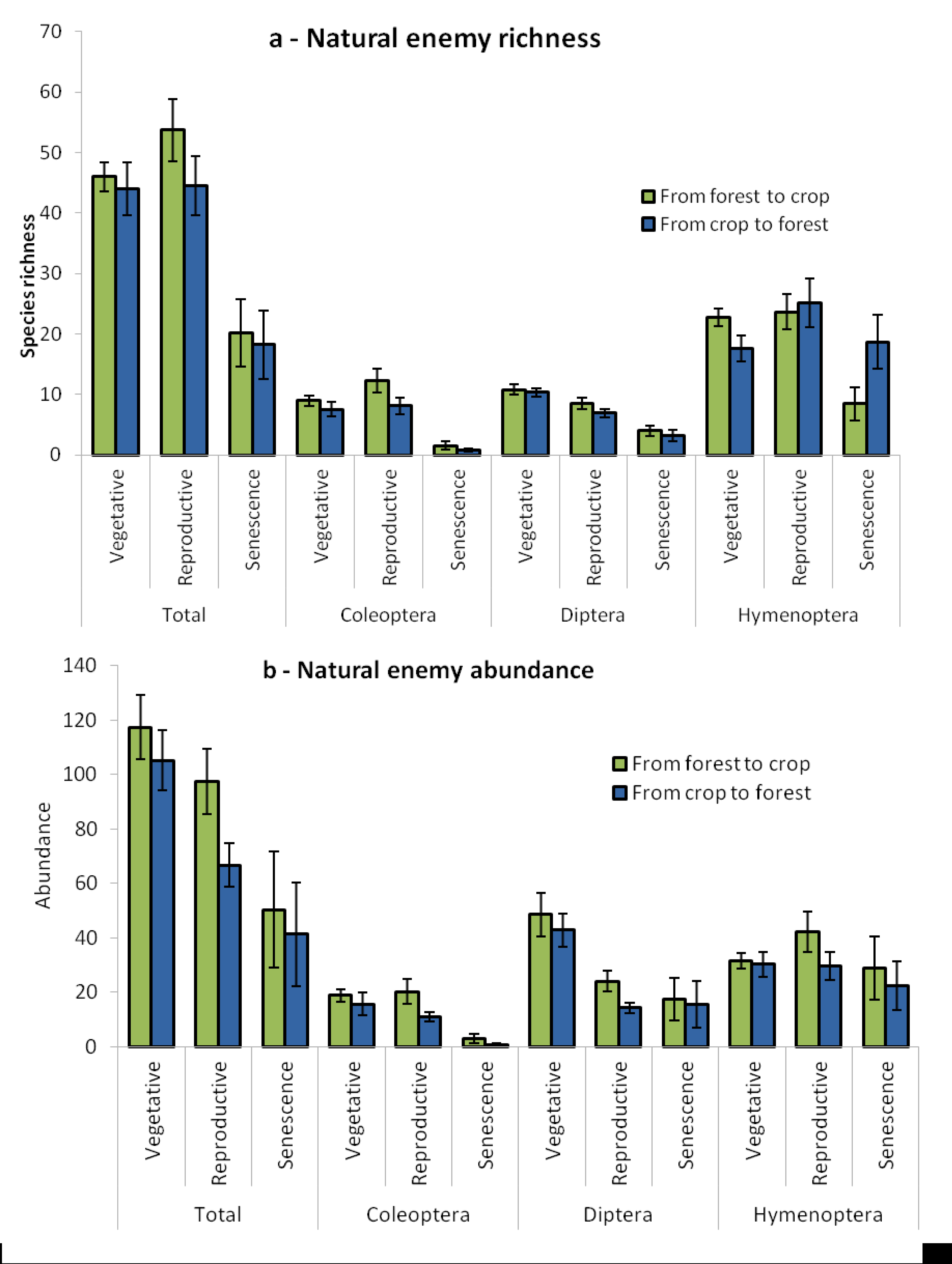

Supplement: S3 Fig — Richness (a) and abundance (b) of total natural enemies and their three main orders (Coleoptera, Diptera and Hymenoptera) moving toward crops (in green) and towards forest (in blue) at soybean phenological phases: vegetative, reproductive and senescence. (TIF) [file pone.0158836.s003.tif]

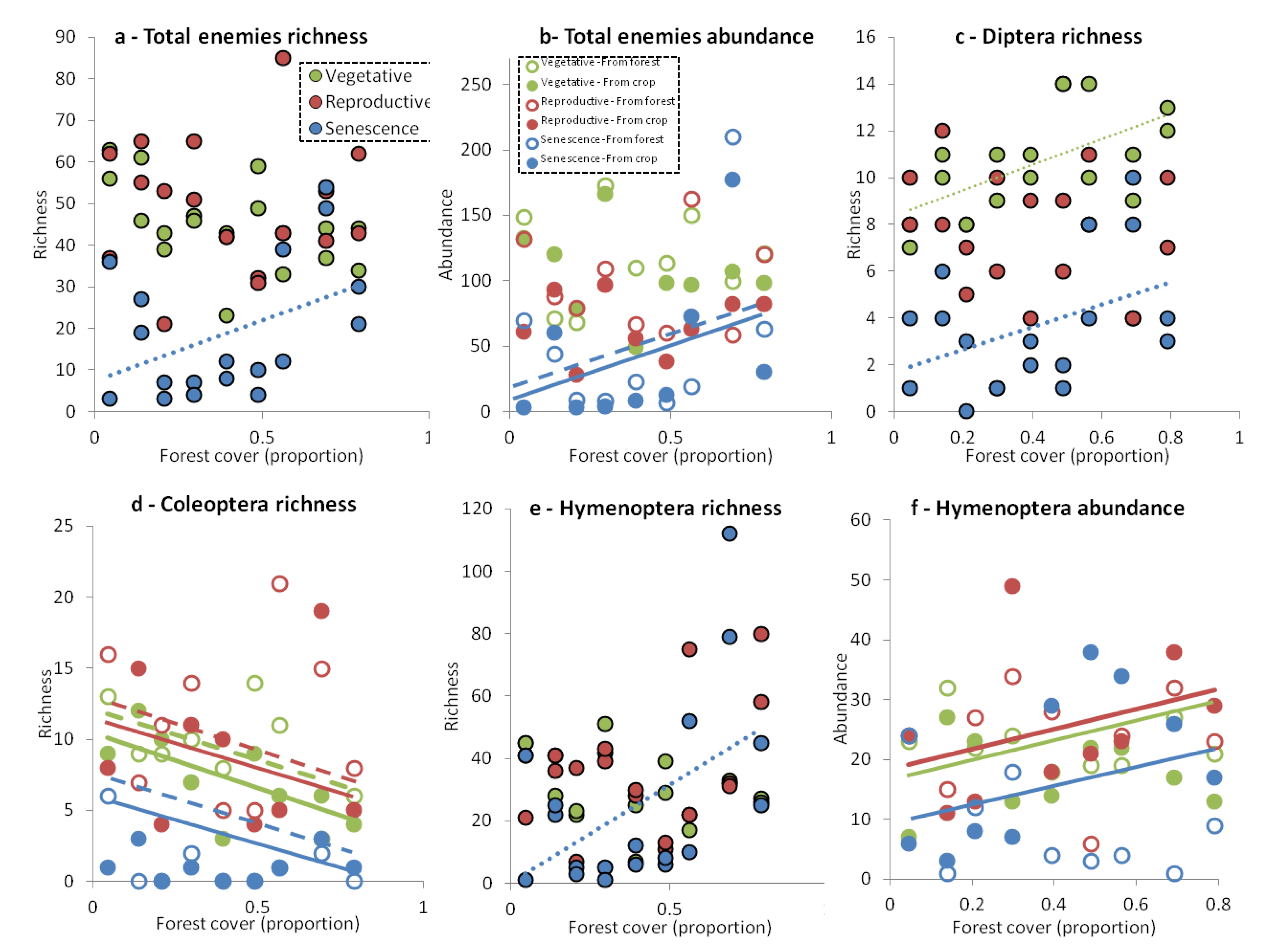

Supplement: S4 Fig — Significant relationships between proportion of forest cover in the landscape and movement of natural enemies at soybean phenological phases: vegetative (green), reproductive (red) and senescence (blue). When differences between movement directions were significant, empty circles represent movement from the forest and filled circles from the crop. Lines are used only when the relation with forest cover was significant, with dashed lines for movement from the forest, solid lines for movement from the crop and dotted lines for both directions. (a) Total enemy richness. (b) Total enemy abundance. (c) Diptera richness. (d) Coleoptera richness. (e) Hymenoptera richness. (f) Hymenoptera abundance. (TIF) [file pone.0158836.s004.tif]

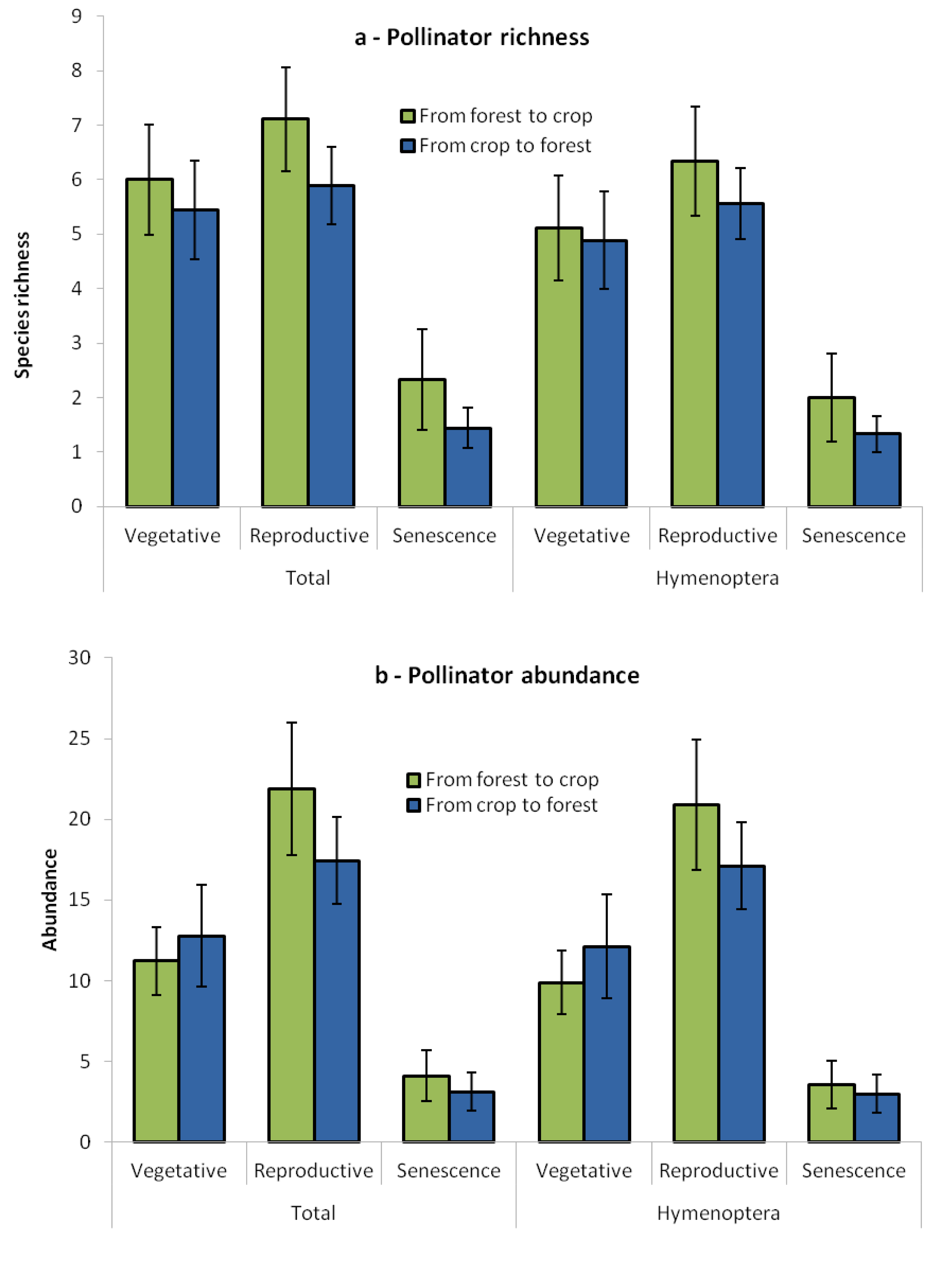

Supplement: S5 Fig — Richness (a) and abundance (b) of total and hymenopteran pollinators moving towards crops (in green) and towards forest (in blue) at soybean phenological phases: vegetative, reproductive and senescence. (TIF) [file pone.0158836.s005.tif]

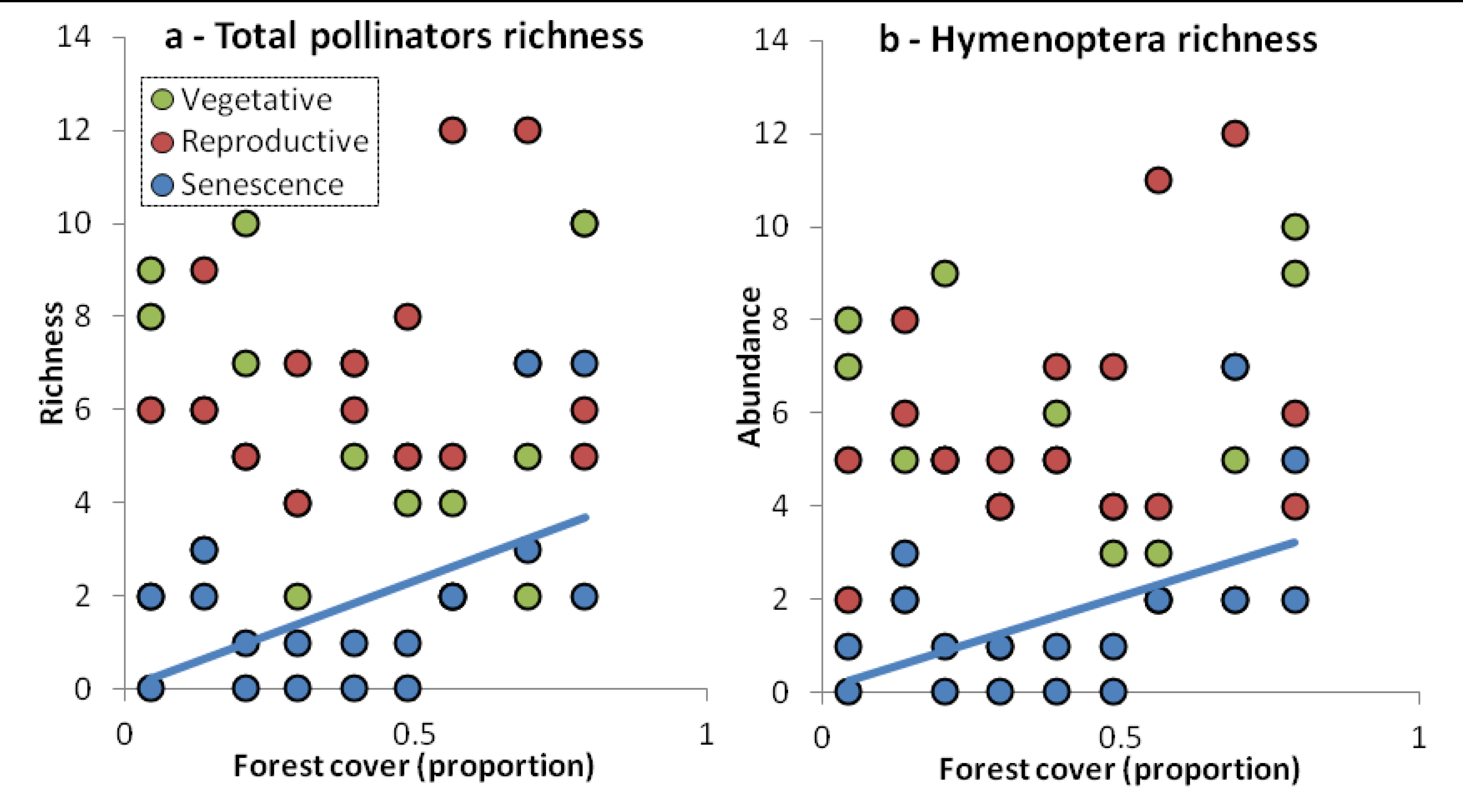

Supplement: S6 Fig — Significant relationships between forest cover in the landscape and movement of pollinator insects at soybean phenological phases: vegetative (green), reproductive (red) and senescence (blue). Lines are used only when the relation with forest cover was significant. (a) Total pollinator richness. (b) Hymenoptera richness. (TIF) [file pone.0158836.s006.tif]

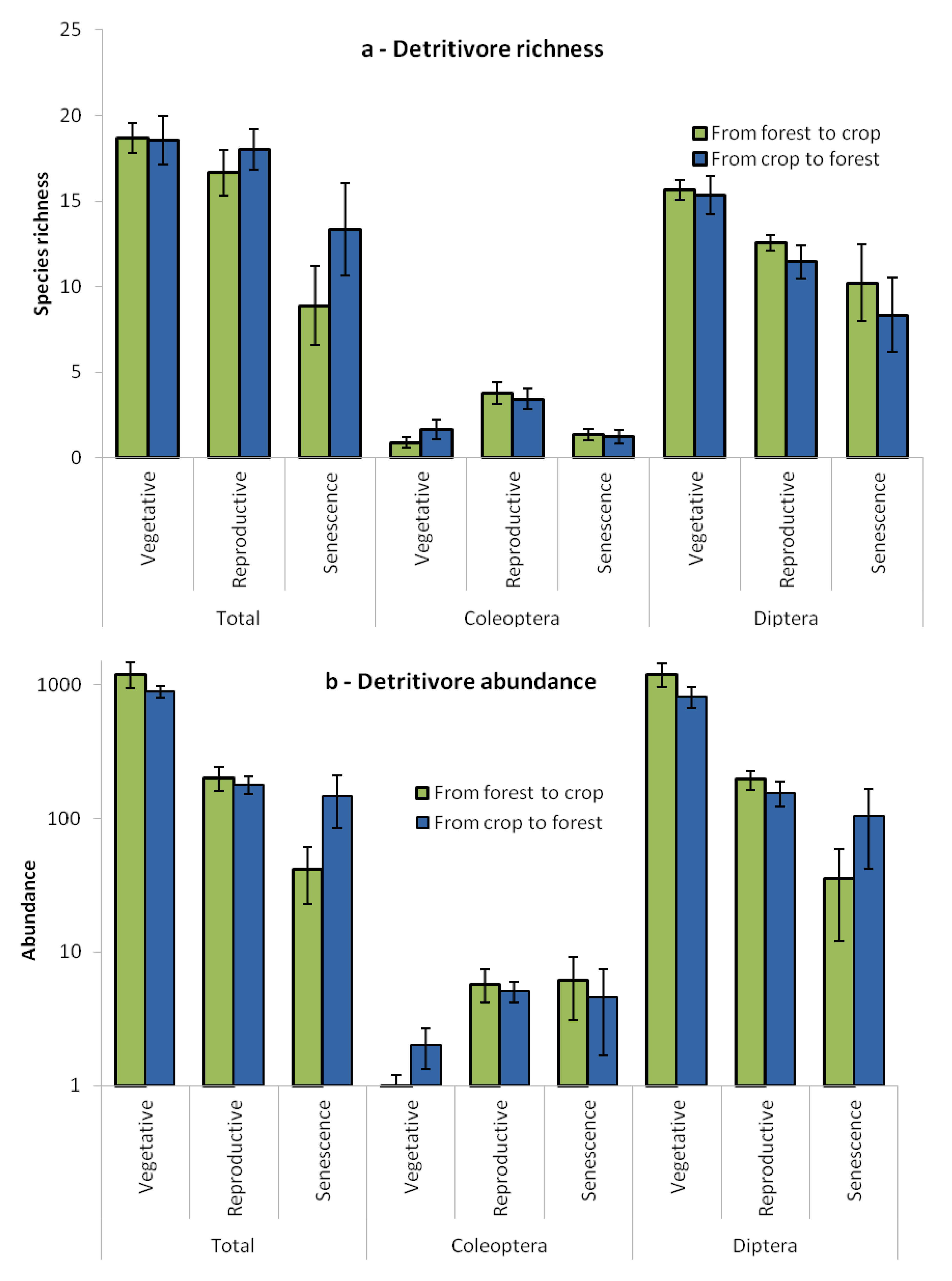

Supplement: S7 Fig — Richness (a) and abundance (b) of total detritivores and the two main orders (Coleoptera, and Diptera) moving towards crops (in green) and towards forest (in blue) at soybean phenological phases: vegetative, reproductive and senescence. (TIF) [file pone.0158836.s007.tif]

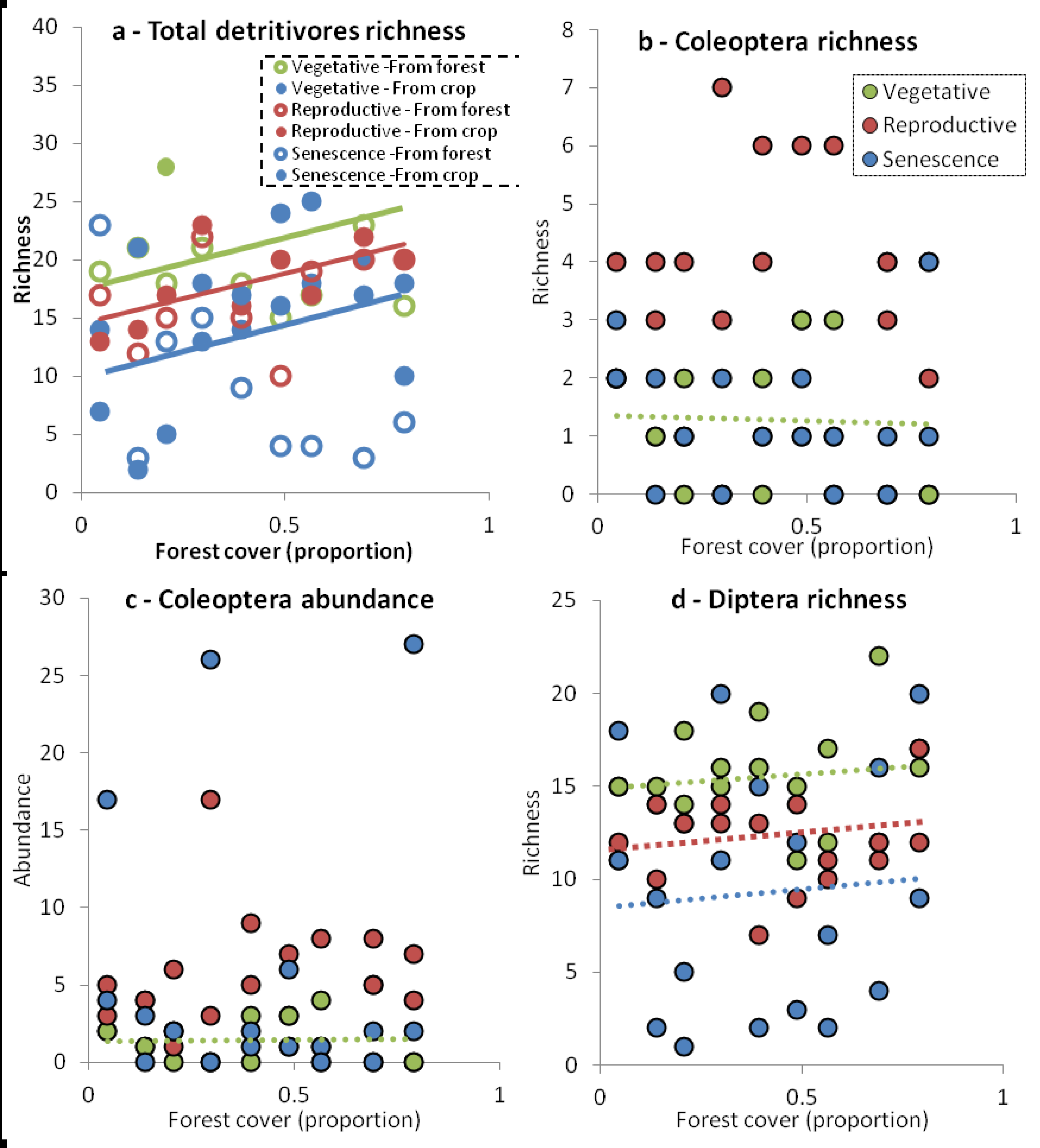

Supplement: S8 Fig — Significant relationships between forest cover in the landscape and movement of detritivore insects at soybean phenological phases: vegetative (green), reproductive (red) and senescence (blue). When differences between movement directions were significant, empty circles represent movement from the forest and filled circles from the crop. Lines are used only when the relation with forest cover was significant, with dashed lines for movement from the forest, solid lines for movement from the crop and dotted lines for both directions. (a) Total detritivore richness. (b) Coleoptera richness. (c) Coleoptera abundance. (d) Diptera richness. (TIF) [file pone.0158836.s008.tif]
