# Supplementary material for: A Moveable Feast: Insects Moving at the Forest-Crop Interface Are Affected by Crop Phenology and the Amount of Forest in the Landscape
Source: PLoS One. 2016 Jul 6;11(7):e0158836. doi: 10.1371/journal.pone.0158836 (PMC4934915; doi:10.1371/journal.pone.0158836)
Supplement: S1 Table — For each functional group and order, the best three models and the full model (which includes all independent variables and their paired interactions) are ind1icated. AICc values for every model and ΔAICc (difference in AICc between each model and the top model) are also included. x indicates interactions between two variables. Independent variables abbreviations: ForestCov = Forest Cover in the landscape; CropPhen = Soybean Crop Phenology; MovDir = Movement Direction. (DOCX) [file pone.0158836.s009.docx]

Table S1 – Summary of model selection by AICc. For each functional group and order, the best three models and the full model (which includes all independent variables and their paired interactions) are indicated. AICc values for every model and ΔAICc (difference in AICc between each model and the top model) are also included. x indicates interactions between two variables.

Independent variables abbreviations: ForestCov = Forest Cover in the landscape; CropPhen = Soybean Crop Phenology; MovDir = Movement Direction.

| Functional Group | Response Variable | Model | AICc | ΔAICc |
| --- | --- | --- | --- | --- |
| Herbivores | Herbivores richness | S_h_ ~ CropPhen  S_h_ ~ ForestCov + CropPhen  S_h_ ~ ForestCov + CropPhen + MovDir  Full Model | 434.8  435.3  437.8  449.9 | -  0.5  3.0  15.1 |
|  | Herbivores abundance | N_h_ ~ ForestCov x CropPhen  N_h_ ~ ForestCov + CropPhen  N_h_ ~ ForestCov x CropPhen +MovDir  Full Model | 747.8  747.8  750.7  759.6 | -  0  2.9  11.8 |
|  | Coleoptera richness | S_hc_ ~ CropPhen  S_hc_ ~ CropPhen + MovDir  S_hc_ ~ CropPhen x MovDir  Full Model | 257.9  260.1  261.7  269.7 | -  2.2  3.8  11.8 |
|  | Coleoptera abundance | N_hc_ ~ ForestCov x CropPhen  N_hc_ ~ CropPhen  N_hc_ ~ ForestCov x CropPhen  Full Model | 374.1  374.1  376.8  390.4 | -  0  2.7  16.3 |
|  | Hemiptera richness | S_hh_ ~ CropPhen  S_hh_ ~ ForestCov + CropPhen  S_hh_ ~ ForestCov x CropPhen  Full Model | 369.1  369.3  370.3  378.9 | -  0.2  1.2  9.8 |
|  | Hemiptera abundance | N_hh_ ~ CropPhen  N_hh_ ~ ForestCov + CropPhen  N_hh_ ~ ForestCov x CropPhen  Full Model | 450.3  451.9  454.2  465.2 | -  1.6  3.9  14.9 |
|  | Lepidoptera richness | S_hl_ ~ MovDir x CropPhen  S_hl_ ~ CropPhen  S_hl_ ~ MovDir + CropPhen  Full Model | 276.0  276.1  276.5  283.7 | -  0.1  0.5  7.7 |
|  | Lepidoptera abundance | N_hl_ ~ ForestCov x CropPhen  N_hl_ ~ CropPhen  N_hl_ ~ ForestCov x CropPhen + MovDir  Full Model | 455.6  456.2  457.6  466.5 | -  0.6  2.0  10.9 |
| Natural enemies | Natural enemies richness | S_e_ ~ ForestCov x CropPhen  S_e_ ~ ForestCov x CropPhen + MovDir  S_e_ ~ CropPhen  Full Model | 464.3  465.5  465.7  486.3 | -  1.2  1.4  22.0 |
|  | Natural enemies abundance | N_e_ ~ ForestCov x CropPhen + MovDir1  N_e_ ~ CropPhen  N_e_ ~ ForestCov x CropPhen  Full Model | 570.2  570.7  571.1  579.1 | -  0.5  0.9  8.9 |
|  | Coleoptera richness | S_ec_ ~ ForestCov + CropPhen + MovDir  S_ec_ ~ ForestCov + CropPhen x MovDir  S_ec_ ~ CropPhen + MovDir  Full Model | 269.5  270.2  270.5  279.3 | -  0.7  1.0  9.8 |
|  | Coleoptera abundance | N_ec_ ~ CropPhen + MovDir  N_ec_ ~ ForestCov + CropPhen x MovDir  N_ec_ ~ CropPhen x MovDir  Full Model | 342.2  343.5  344.2  352.3 | -  1.3  2.0  10.1 |
|  | Diptera richness | S_ed_ ~ ForestCov x CropPhen  S_ed_ ~ ForestCov x CropPhen + MovDir  S_ed_ ~ ForestCov + CropPhen  Full Model | 260.7  261.9  262.8  270.3 | -  1.2  2.1  9.6 |
|  | Diptera abundance | N_ed_ ~ CropPhen  N_ed_ ~ ForestCov x CropPhen + MovDir  N_ed_ ~ ForestCov + CropPhen  Full Model | 459.3  459.8  460.0  468.3 | -  0.5  0.7  9.0 |
|  | Hymenoptera richness | S_ey_ ~ ForestCov x CropPhen + MovDir x CropPhen  S_ey_ ~ ForestCov + CropPhen + MovDir  Full Model  S_ey_ ~ ForestCov x CropPhen + MovDir | 410.1  415.9  416.2  420.7 | -  5.8  6.1  10.6 |
|  | Hymenoptera abundance | N_ey_ ~ ForestCov x CropPhen + MovDir  N_ey_ ~ ForestCov x CropPhen  N_ey_ ~ CropPhen  Full Model | 474.8  474.8  476.9  483.1 | -  0  2.1  8.3 |
| Detritivores | Detritivores richness | S_d_ ~ ForestCov x MovDir + CropPhen  S_d_ ~ MovDir + CropPhen  S_d_ ~ ForestCov x MovDir + CropPhen  Full Model | 355.2  355.4  356.0  362.5 | -  0.2  0.8  7.3 |
|  | Detritivores abundance | N_d_ ~ MovDir x CropPhen  N_d_ ~ MovDir x CropPhen + ForestCov  N_d_ ~ MovDir + CropPhen + ForestCov  Full Model | 720.8  723.7  724.9  729.0 | -  2.9  4.1  8.2 |
|  | Coleoptera richness | S_dc_ ~ ForestCov x CropPhen  S_dc_ ~ CropPhen  S_dc_ ~ ForestCov + CropPhen  Full Model | 185.6  187.7  188.3  195.1 | -  2.1  2.7  9.5 |
|  | Coleoptera abundance | N_dc_ ~ ForestCov x CropPhen  N_dc_ ~ CropPhen  N_dc_ ~ ForestCov + CropPhen  Full Model | 267.3  268.4  272.2  279.6 | -  1.1  4.9  12.3 |
|  | Diptera richness | S_dd_ ~ ForestCov + CropPhen  S_dd_ ~ ForestCov + CropPhen + MovDir  S_dd_ ~ CropPhen  Full Model | 327.8  329.0  329.3  337.2 | -  1.2  1.5  9.4 |
|  | Diptera abundance | N_dd_ ~ CropPhen  N_dd_ ~ ForestCov + CropPhen  N_dd_ ~ ForestCov + CropPhen + MovDir  Full Model | 715.1  715.9  718.3  726.7 | -  0.8  3.2  11.6 |
| Pollinators | Pollinators richness | S_p_ ~ ForestCov x CropPhen  S_p_ ~ ForestCov x CropPhen + MovDir  S_p_ ~ ForestCov x MovDir + ForestCov x CropPhen  Full Model | 244.3  244.8  247.5  252.9 | -  0.5  3.15  8.6 |
|  | Pollinators abundance | N_p_ ~ CropPhen  N_p_ ~ ForestCov + CropPhen  N_p_ ~ ForestCov + CropPhen + MovDir  Full Model | 357.6  358.0  360.3  372.4 | -  0.4  2.7  14.8 |
|  | Hymenoptera richness | S_py_ ~ ForestCov x CropPhen  S_py_ ~ ForestCov x CropPhen + MovDir  S_py_ ~ ForestCov + CropPhen  Full Model | 237.3  239.1  239.2  247.4 | 0  1.8  1.9  10.1 |
|  | Hymenoptera abundance | N_py_ ~ CropPhen  N_py_~ ForestCov + CropPhen  N_py_ ~ CropPhen  Full Model | 391.8  394.3  396.9  405.3 | 0  2.5  5.1  13.5 |
